# A randomised comparison of deferasirox *versus* deferoxamine for the treatment of transfusional iron overload in sickle cell disease

**DOI:** 10.1111/j.1365-2141.2006.06455.x

**Published:** 2007-02-01

**Authors:** Elliott Vichinsky, Onyinye Onyekwere, John Porter, Paul Swerdlow, James Eckman, Peter Lane, Beatrice Files, Kathryn Hassell, Patrick Kelly, Felicia Wilson, Françoise Bernaudin, Gian Luca Forni, Iheanyi Okpala, Catherine Ressayre-Djaffer, Daniele Alberti, Jaymes Holland, Peter Marks, Ellen Fung, Roland Fischer, Brigitta U Mueller, Thomas Coates

**Affiliations:** 1 Children's Hospital and Research Center Oakland, CA; 2 Howard University Washington, DC, USA; 3 University College Hospital London, UK; 4 Wayne State University School of Medicine Detroit, MI; 5 Emory University School of Medicine Atlanta, GA; 6 Children's Healthcare Atlanta, GA; 7 University of Colorado Health Sciences Center Denver, CO; 8 Children's Hospital Medical Center Cincinnati, OH; 9 University of South Alabama Medical Center Mobile, AL, USA; 10 Department of Paediatrics, Centre Hospitalier Intercommunal Créteil, France; 11 Centro della Microcitemia, Ospedale Galliera Genoa, Italy; 12 St Thomas Hospital London, UK; 13 Novartis Pharmaceuticals Corporation, East Hanover NJ, USA; 14 University Medical Centre Hamburg-Eppendorf Germany; 15 Texas Children's Hospital, Baylor College of Medicine Houston, TX; 16 Children's Hospital Los Angeles, Los Angeles CA, USA

**Keywords:** deferasirox, ICL670, Exjade, sickle cell disease, iron overload

## Abstract

Deferasirox is a once-daily, oral iron chelator developed for treating transfusional iron overload. Preclinical studies indicated that the kidney was a potential target organ of toxicity. As patients with sickle cell disease often have abnormal baseline renal function, the primary objective of this randomised, open-label, phase II trial was to evaluate the safety and tolerability of deferasirox in comparison with deferoxamine in this population. Assessment of efficacy, as measured by change in liver iron concentration (LIC) using biosusceptometry, was a secondary objective. A total of 195 adult and paediatric patients received deferasirox (*n* = 132) or deferoxamine (*n* = 63). Adverse events most commonly associated with deferasirox were mild, including transient nausea, vomiting, diarrhoea, abdominal pain and skin rash. Abnormal laboratory studies with deferasirox were occasionally associated with mild non-progressive increases in serum creatinine and reversible elevations in liver function tests. Discontinuation rates from deferasirox (11·4%) and deferoxamine (11·1%) were similar. Over 1 year, similar dose-dependent LIC reductions were observed with deferasirox and deferoxamine. Once-daily oral deferasirox has acceptable tolerability and appears to have similar efficacy to deferoxamine in reducing iron burden in transfused patients with sickle cell disease.

The majority of patients with sickle cell anaemia have received repeated blood transfusions by adulthood (Styles & Vichinsky, 1994; Wanko & Telen, 2005). Transfusion therapy is likely to further increase in paediatric patients because of recent evidence indicating its ability to prevent organ injury and improve the outcome of complications (Adams *et al*, 1998; The Optimizing Primary Stroke Prevention in Sickle Cell Anemia (STOP2) Trial Investigators, 2005). However, because the body has no physiological mechanism to actively excrete excess iron, repeated blood transfusions lead to increased body iron burden, including iron deposition into the liver, heart and endocrine organs (McLaren *et al*, 1983). While studies are limited, progressive iron loading and subsequent tissue injury in sickle cell disease appears similar to other transfused populations (Ballas, 2001; Manci *et al*, 2003; Vichinsky *et al*, 2005). Guidelines from consensus conferences have therefore recommended daily treatment with deferoxamine in iron-overloaded patients (National Institutes of Health, 2002).

Effective administration of iron chelation therapy has been limited by the route of its administration. Although deferoxamine is effective in removing iron from the body, due to very poor oral bioavailability and a short half-life it must be administered by subcutaneous or intravenous infusion, usually over 8–12 h on 5–7 d/week (Porter, 2001). Compliance with this regimen is often poor (Treadwell *et al*, 2005). The availability of a once-daily, oral alternative would potentially facilitate improved compliance, and thereby reduce morbidity and mortality from iron overload.

Deferasirox is an orally absorbed iron chelator that has been developed for the management of transfusional iron overload. Its safety, tolerability and efficacy in reducing body iron burden have been demonstrated in patients with *β*-thalassaemia major and in other chronic transfusion-dependent anaemias (Cappellini *et al*, 2006). Over a 1-year period, deferasirox was demonstrated to maintain or reduce body iron burden in regularly transfused patients who had baseline liver iron concentration (LIC) values of at least 7 mg Fe/g dry weight (dw) with an acceptable side effect profile.

Individuals with sickle cell disease often have abnormal baseline renal function. Therefore, it was of note that preclinical studies conducted with deferasirox in animals indicated that a potential target organ of toxicity was the kidney. High doses of deferasirox were found to be associated with renal tubular damage in rats and marmosets, particularly in those that did not have iron overload (Vanorden & Hagemann, 2006). This effect was felt most likely, but not definitively, to be related to the pharmacological effect of deferasirox chelating iron. Based on these preclinical data, simple extrapolation of the safety profile of deferasirox from a population of patients with *β*-thalassaemia major to individuals with sickle cell disease was not deemed to be appropriate. In addition, whether or not treatment with deferasirox might trigger or exacerbate sickle cell crises was unknown. Thus, the primary objective of this randomised, open-label, phase II trial was to evaluate the safety and tolerability of deferasirox in comparison with deferoxamine in this population.

## Methods

### Study design and patient population

#### Patient eligibility

Patients with sickle cell disease ≥2 years of age and with iron overload from repeated blood transfusions were enroled in this trial. Patients receiving regular blood transfusions or those sporadically transfused who received at least 20 units of packed red blood cells or equivalent were eligible. Prior chelation therapy was permitted but was not mandatory. The serum ferritin level for entry into the screening period of this study was ≥1000 *μ*g/l. Patients eligible for entry into the screening period subsequently had biosusceptometry performed (see below) in order to determine that they had a sufficiently high body iron burden to be eligible for treatment on the trial.

Patients were excluded if they had a serum creatinine above the upper limit of normal (ULN), if they had significant proteinuria (as indicated by a urinary protein:creatinine ratio of ≥0·5 confirmed at two visits), or if they had active hepatitis B or C. Active hepatitis B was defined as liver function tests above the normal range, together with a positive antigen (hepatitis B e antigen, hepatitis B surface antigen) test or positive IgM core antibody test in conjunction with a negative hepatitis B surface antibody test. Active hepatitis C was defined as liver function tests above the normal range in the presence of a positive hepatitis C antibody test and detectable hepatitis C RNA levels. Other exclusion criteria were second and third atrioventricular block, QT interval prolongation, or therapy with digoxin or similar medications. Treatment with *β*-blockers or angiotensin-converting enzyme inhibitors was permitted. Patients with chelation therapy-associated ocular toxicity were excluded.

After screening requirements were met and informed consent was obtained, eligibility based on the presence of iron overload was confirmed by non-invasive determination of LIC using Superconducting QUantum Interference Device (SQUID) biosusceptometry (Brittenham *et al*, 1982). Patients receiving simple transfusions needed to have a LIC of ≥2 mg Fe/g dw, and those receiving exchange transfusions needed to have a LIC of ≥5 mg Fe/g dw at study entry. Biosusceptometry was performed at three centres under a standardised operational procedure (Turin, Italy; Hamburg, Germany; Oakland, USA). For consistency, all SQUID biosusceptometry assessments for a given subject were conducted at the same site.

*In vivo* LIC values by SQUID biosusceptometry (approximately wet weight) were converted into dry weight LIC values using the widely adopted wet-to-dry weight ratio of 3·33 (Olivieri & Brittenham, 1997). Studies performed as part of another clinical trial that was initiated just before the current trial and completed prior to it found that a more precise conversion factor of twice the adopted factor was applicable to all three sites (Fischer *et al*, 2006). Because of these findings, adjusted LIC values are presented unless otherwise noted.

#### Randomisation

Patients were randomised to receive deferasirox or deferoxamine in a 2:1 manner. The randomisation was performed using an interactive voice response system and was stratified according to the following age groups: 2 to <6 years, 6 to <12 years, 12 to <16 years and 16 years and older. The randomisation sequence included permuted block groups of six patients for each of the three age strata.

#### Treatment plan

The study duration was 52 weeks. The initial 24 patients enroled were randomised to receive deferasirox 10 mg/kg or deferoxamine at recommended doses of 20–60 mg/kg based on initial LIC. Subsequently, additional safety information became available for deferasirox suggesting a need to modify the starting dose (Cappellini *et al*, 2006). Therefore, following the enrolment of the first 24 patients, the study was amended so that all subsequent patients randomised to deferasirox were dosed at 10–30 mg/kg according to baseline LIC. Deferasirox was given once daily each morning as a dispersed solution in water, half-an-hour before breakfast. The dose of deferasirox was reduced by one dose level and not re-escalated for patients 15 years and older if serum creatinine increased >33% above baseline on two consecutive occasions. For children less than 15 years of age, the dose was only decreased if these values were also above the age-appropriate ULN. Deferasirox was interrupted for moderate or severe skin rash and reinstituted at half the initial dose, and dose re-escalation was permitted. Deferoxamine was administered as a slow subcutaneous infusion over 8–12 h using electronic Microject Chrono® (Medical Technology, Turin, Italy) infusion pumps on 5–7 d a week. In order to facilitate comparison of different schedules, all deferoxamine doses reported were normalised to administration for 5 d/week (i.e. 50 mg/kg administered 7 d/week would be reported as 70 mg/kg).

The trial was conducted in accordance with the Declaration of Helsinki. Institutional Review Board approval was obtained at each participating institution and written informed consent was obtained from all patients or guardians prior to participation in any study procedures. Novartis Pharmaceuticals Corporation (East Hanover, NJ, USA) coordinated the design and execution of this trial and contributed to the analysis and interpretation of the trial data. Novartis Pharmaceuticals Corporation also collaborated with the external authors to assist in the development and approval of the manuscript for publication.

#### Safety assessments

Laboratory assessments were performed at least monthly and included complete blood counts with differential counts. Biochemistry testing included electrolytes, glucose, liver function tests, gamma-glutaryl-transferase, lactate dehydrogenase, cholesterol, triglycerides, uric acid, total protein, C-reactive protein, copper and zinc levels. Iron parameters included total iron, transferrin, transferrin saturation and ferritin. Urinary testing performed on random collections included determination of creatinine, total protein and albumin. Physical examinations, electrocardiograms (ECGs), audiometry and ophthalmological tests were performed at baseline, 12, 24, 36 and 52 weeks. In patients less than 16 years of age, additional assessments included growth velocity and pubertal stage.

#### Efficacy assessments

Liver iron concentration was determined by SQUID biosusceptometry at baseline, 24 and 52 weeks. The 24-week assessment was performed primarily for safety purposes, and the change in LIC was calculated between baseline and 52 weeks. Serum ferritin was assessed monthly during the study and the change was determined using the baseline and final ferritin level.

#### Compliance

For deferasirox, compliance was assessed by counting the number of tablets returned in bottles at each visit. For deferoxamine, the numbers of vials returned at each visit were counted.

### Statistical analysis

The data were analysed under supervision of the trial statistician and were reviewed by the investigators. The sample size was calculated based on the number of deferasirox-treated patients required to detect adverse experiences having an underlying event rate of at least 4% with 95% confidence, according to the method of Hanley and Lippman-Hand (1983). The assessment of safety was based mainly on the frequency of adverse events and on the number of laboratory values that fell outside the predetermined ranges. Other safety data (e.g. ECGs, vital signs and other tests) were considered as appropriate. All safety data are reported for patients who received at least one dose of study medication.

The main efficacy endpoint was the change in LIC from baseline assessed at 52 weeks, adjusted according to the transfusion category (simple, exchange, simple and exchange). Descriptive statistics are presented throughout where appropriate. Testing for statistical significance for differences between baseline and end-of-study for each treatment group was performed using Student's *t*-test. Using a two-sided test, *P*-values <0·05 were considered to be statistically significant.

## Results

### Study population

Patients were recruited by investigators at 44 sites in the USA, France, Italy, UK and Canada. Of the 203 eligible patients randomised, 195 received treatment and are included in the safety population. The percentage of patients discontinuing deferasirox and deferoxamine was similar (11·4% vs. 11·1% respectively). Adverse events resulted in discontinuations in 5·3% of patients on deferasirox and 3·2% of patients on deferoxamine. The remainder of the discontinuations was due to patients lost to follow-up, withdrawal of consent and protocol violations. Six patients randomised to deferasirox and one patient randomised to deferoxamine withdrew consent. The reasons for withdrawal of consent were not included in the database. Three patients randomised to deferoxamine were unwilling to comply with drug administration and were discontinued due to protocol violations. Two patients randomised to deferasirox and one patient randomised to deferoxamine could not be located for follow-up. The two groups were well balanced for age, transfusion type and history of prior chelation therapy (Table I). Baseline LIC and ferritin values in the two groups were similarly elevated.

**Table I:** Patient demographics and selected clinical parameters.

Variable/statistic	Deferasirox (*n* = 132)	Deferoxamine (*n* = 63)	All patients (*n* = 195)
Age, years			
Median	15	16	15
Range	3–54	3–51	3–54
Age group, %			
<6 years	3·0	4·8	3·6
6 to <12 years	22·7	23·8	23·1
12 to <16 years	25·0	20·6	23·6
16 to <50 years	47·7	49·2	48·2
50 to <65 years	1·5	1·6	1·5
Sex, %			
Female	60·6	55·6	59·0
Race, %			
Caucasian	6·1	4·8	5·6
Black	89·4	93·7	90·8
Others	4·5	1·6	3·6
Ferritin, *μ*g/l			
Median	3460	2834	3298
Min–max	1082–12 901	1015–15 578	1015–15 578
Baseline ALT, %			
≤2·5 ULN	83·3	92·1	86·2
>2·5 ULN	15·9	7·9	13·3
Missing	0·8	–	0·5
History of hepatitis B and/or C, %			
Present	7·6	6·3	7·2
Prior chelation therapy, %			
Deferoxamine or deferiprone	62·9	60·3	62·1
Blood transfusions during study (units of packed red blood cells)			
Median	12	12	12
Range	0–24	1–22	0–24

ALT, alanine aminotransferase; ULN, upper limit of normal.

### Drug administration and compliance

The doses of deferasirox and deferoxamine were based on the baseline LIC determined by SQUID biosusceptometry converted by the adopted wet-to-dry weight factor of 3·33. The percentage of patients falling into each dose category was similar for the two treatment arms (Table II). The mean doses of deferasirox administered were approximately half those of deferoxamine. During the study the ratios of the administered to intended doses of therapy were high (1·16 for deferasirox and 0·97 for deferoxamine), indicating high adherence to the prescribed treatment regimens.

**Table II:** Dosing algorithm according to baseline liver iron concentration (LIC) groups (reported and adjusted) and average daily doses administered.

	Baseline LIC (mg Fe/g dw)			
	
Baseline LIC group	≤3	>3–7	>7–14	>14
*Deferasirox* (*n* = 132)	(*n* = 4)	(*n* = 64)	(*n* = 46)	(*n* = 18)
Protocol assigned dose	5 mg/kg	10 mg/kg	20 mg/kg	30 mg/kg
Reported mean LIC ± SD*	2·5 ± 0·4	7·9 ± 5·5	9·8 ± 1·9	17·5 ± 3·0
Adjusted mean LIC ± SD	5·0 ± 0·8	15·8 ± 11·0	19·6 ± 3·8	35·0 ± 6·0
Deferasirox dose (mg/kg)†	9·5 ± 3·2	13·0 ± 3·1	19·7 ± 2·1	28·0 ± 2·8
Min–max deferasirox dose	5·0–12·3	8·4–23·9	10·0–24·5	22·8–30·0
*Deferoxamine* (DFO, *n* = 63)	(*n* = 6)	(*n* = 21)	(*n* = 20)	(*n* = 16)
Protocol assigned dose†	20–30 mg/kg	25–35 mg/kg	35–50 mg/kg	≥50 mg/kg
Reported mean LIC ± SD	3·9 ± 3·5	5·2 ± 2·1	8·6 ± 3·0	14·3 ± 5·4
Adjusted mean LIC ± SD	7·8 ± 7·0	10·4 ± 4·2	17·2 ± 6·0	28·6 ± 10·8
DFO dose (mg/kg)‡	22·9 ± 3·9	28·7 ± 3·2	36·6 ± 9·5	50·0 ± 7·3
Min–max DFO dose	20·0–29·5	21·6–34·4	7·0–52·6	32·4–62·0
Deferasirox/DFO dose ratio	1:2·4	1:2·2	1:1·85	1:1·8

* For the reported LIC values a correction factor of 3·33 was used to convert the wet weight to dry weight values (Brittenham *et al*, 1982); for the adjusted values a correction factor of 6·66 was used (Olivieri & Brittenham, 1997).

† Average daily doses are reported for the 1-year period of drug administration. For deferoxamine the reported doses are normalised to those administered with a 5-d treatment regimen.

‡ Patients in the two lower LIC groups (LIC ≤ 3 and >3–7 mg Fe/g dw) who were randomised to treatment with deferoxamine were allowed to continue on their current deferoxamine dosage, even if this was higher than recommended in this table.

### Safety and tolerability

Adverse events, irrespective of the relationship to study medication, which occurred in more than 10% of patients receiving either treatment, are shown in Table III. As arbitrarily defined by an increased frequency of at least 5% indicating a potential relationship to drug administration, adverse events observed more commonly with deferasirox included abdominal pain, nausea, vomiting, diarrhoea, back pain and skin rash. Those observed more commonly in patients receiving deferoxamine included cough, nasopharyngitis and viral infection. A similar incidence of several adverse events, such as headache and upper respiratory tract infection, was observed with both drugs, indicating a possible relationship to the underlying disease or to the administration of both drugs.

**Table III:** Percentage of adverse events reported with greater than 10% frequency in either arm.

Adverse events, %	Deferasirox (*n* = 132)	Deferoxamine (*n* = 63)	All patients (*n* = 195)
Sickle cell anaemia with crisis	33·3	31·7	32·8
Headache	28·8	33·3	30·3
Abdominal pain	28·0	14·3	23·6
Nausea	22·7	11·1	19·0
Pyrexia	21·2	17·5	20·0
Vomiting	21·2	15·9	19·5
Diarrhoea	19·7	4·8	14·9
Back pain	18·2	5·9	17·4
Upper respiratory tract infection	18·2	19·0	18·5
Arthralgia	15·2	14·3	14·9
Pain in extremity	14·4	12·7	13·8
Pharyngolaryngeal pain	14·4	9·5	12·8
Cough	13·6	20·6	15·9
Nasopharyngitis	13·6	20·6	15·9
Rash	13·6	4·8	10·8
Constipation	9·8	14·3	11·3
Chest pain	9·1	12·7	10·3
Viral infection	4·5	11·1	6·7

The gastrointestinal adverse events that patients experienced with deferasirox were generally transient in nature and lasted about 1 week maximum. The number of patients receiving deferasirox and deferoxamine that reported serious adverse events was similar (46·2% and 42·9% respectively) and the most common serious adverse event in both groups was sickle cell anaemia with crisis (33·3% and 31·7% respectively).

Haematological parameters were similar in the deferasirox and deferoxamine groups. Mild, stable increases in serum creatinine were observed in 36·4% and 22·2% of patients receiving deferasirox and deferoxamine respectively. Change in the mean ± SD (range) creatinine from baseline to end-of-study was 6·30 ± 9·00 *μ*mol/l (−8·90 to 39·80 *μ*mol/l) with deferasirox and 3·06 ± 9·43 *μ*mol/l (−17·80 to 22·10 *μ*mol/l) with deferoxamine. The number of patients with serum creatinine values that also exceeded the ULN was similar in the two groups (2·3% and 3·2% respectively). Approximately 30% of patients in both the deferasirox and deferoxamine groups had urinary protein to creatinine ratios of >0·2 mg/mg at baseline, and this percentage did not change measurably in either group over the course of the study.

Two consecutive alanine aminotransferase (ALT) levels >5× ULN were observed in five patients (3·8%) treated with deferasirox and in none treated with deferoxamine. Three of these individuals treated with deferasirox had at least one ALT level >5× ULN during the screening period. The elevations were transient in four patients even with continued drug administration. In one patient, a persistently elevated ALT led to interruption of deferasirox therapy. After normalisation of the ALT level the individual was rechallenged with deferasirox and the elevation in ALT recurred. Treatment was permanently discontinued and the ALT returned to normal.

Paediatric growth across the various age groups of children assessed by growth velocity was similar with deferasirox and deferoxamine. The change in growth velocity in patients <6, 6 to <12 and 12–16 years was 7·1 ± 1·7, 6·0 ± 3·2 and 4·6 ± 4·0 cm/year, respectively, for those treated with deferasirox *versus* 6·1 ± 1·9, 4·8 ± 2·5 and 3·4 ± 3·8 cm/year, respectively, for those treated with deferoxamine. In addition, there were no differences in sexual maturity between patients receiving deferasirox and deferoxamine as assessed by Tanner stage (Tanner, 1978).

### Efficacy

Iron intake was calculated from the total amount of red blood cells transfused as previously described (Cappellini *et al*, 2006). For exchange transfusions, the mg amount of iron removed was calculated based on the volume of packed red blood cells removed (1 ml red blood cells removed equals 1·08 mg iron). The mean and median amount of iron administered in the form of blood transfusions during the study was nearly identical in patients receiving deferasirox and deferoxamine (0·22 mg/kg/d and 0·23 mg/kg/d respectively). The majority of patients (58·5%) received simple transfusions during the course of the study. The remaining patients received either exchange transfusions alone (18·5%), a combination of simple and exchange transfusions (21·0%), or no transfusions (2·1%).

**Fig 1:**
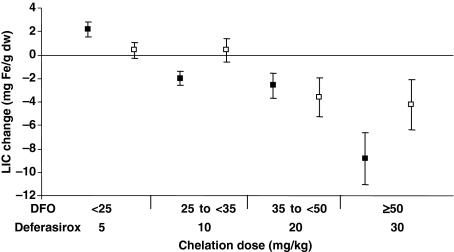
Adjusted change (mean ± SD) in liver iron concentration (LIC) according to assigned treatment category for deferasirox (solid squares) and deferoxamine (DFO, open squares). Statistically significant reductions in LIC were observed with deferasirox in the dose groups treated with 10 (*n* = 60), 20 (*n* = 45) and 30 (*n* = 14) mg/kg with *P*-values of 0·001, 0·014, and <0·001 respectively.

Administration of deferasirox resulted in a statistically significant reduction in LIC for the overall population, adjusted for transfusion category, of −3·0 ± 6·2 mg Fe/g dw (*P* < 0·001). This was similar to the reduction observed with deferoxamine for the overall population, adjusted for transfusion category, of −2·8 ± 10·4 mg Fe/g dw (*P* = 0·022). Statistically significant reductions in LIC at the level of *P* < 0·05 were observed with treatment at deferasirox doses of 10–30 mg/kg and at a deferoxamine dose of 35 to <50 mg/kg (Fig 1). Similar reductions in LIC occurred across all age groups of patients enroled into the trial.

Comparing patients who were categorised as only receiving simple transfusions to those only receiving exchange transfusions, the reductions in LIC observed with deferasirox were −1·6 ± 5·78 mg Fe/g dw (*n* = 62) and −6·6 ± 5·60 mg Fe/g dw (*n* = 22) respectively. The corresponding reductions in patients receiving deferoxamine were −1·4 ± 3·12 mg Fe/g dw (*n* = 35) and −1·4 ± 3·90 (*n* = 10) respectively.

**Fig 2:**
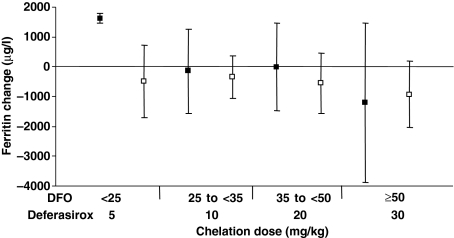
Change (mean ± SD) in serum ferritin according to assigned baseline liver iron concentration (LIC) category for deferasirox (solid squares) and deferoxamine (DFO, open squares).

Changes in serum ferritin between baseline and end-of-study were dose dependent and generally paralleled the changes in LIC observed (Fig 2). However, there was notable intra-patient variability in this parameter during the study. In the overall patient populations treated with deferasirox and deferoxamine, the changes observed were −183 ± 1651 *μ*g/l and −558 ± 951 *μ*g/l respectively.

## Discussion

Sickle cell disease results in acute complications and progressive multi-organ failure. Transfusion therapy reduces the number of erythrocytes containing sickle haemoglobin and reduces the vascular complications associated with the disease. In the STOP trial, which recruited asymptomatic children at risk for cerebral infarction, transfusions resulted in a 90% reduction in the rate of first stroke (Adams *et al*, 1998). In addition to preventing primary and secondary stroke in children, preventative transfusion programmes decrease the frequency of total hospitalisations, vasoocclusive events, acute chest syndrome and growth failure (Vichinsky *et al*, 2005). Chronic transfusions have been recommended in order to decrease the morbidity of end-organ failure associated with heart, pulmonary and renal disease in children and adults (National Institutes of Health, 2002).

Despite the significant benefits that such transfusions may bring, they result in iron overload that is clearly associated with morbidity and mortality in *β*-thalassaemia (Gabutti & Piga, 1996; Schrier & Angelucci, 2005). Evidence is also accumulating regarding the deleterious effects of iron overload in patients with sickle cell disease. This transfusion-related complication is estimated to affect a sizable number of sickle cell patients (Hagar & Vichinsky, 2000). As indicated by the baseline LIC and serum ferritin levels of patients enroled into this clinical trial, patients with sickle cell disease receiving blood transfusions may have markedly elevated iron burdens. These levels are similar to those commonly encountered in *β*-thalassaemia major and may place them at risk for serious complications.

This comparative study demonstrated that deferasirox was reasonably well tolerated in patients with sickle cell disease who have relatively normal renal and hepatic function. There was no excess in sickle cell crises or infections when compared with deferoxamine. Although the absolute magnitude of the reduction may be under- or overestimated because biosusceptometry using SQUID, rather than liver biopsy, was utilised in order to determine the change in LIC, similar reductions in LIC were observed with deferasirox and deferoxamine in the overall populations and across all age groups. As would be expected, greater reductions in LIC were observed with deferasirox in patients receiving exchange transfusions. A similar trend was not observed with deferoxamine, possibly reflecting the relatively small sample size. Changes in serum ferritin were somewhat more variable than those observed in LIC, as would be expected for this parameter in patients with sickle cell disease (Brittenham *et al*, 1993).

The most notable adverse events reported were transient gastrointestinal symptoms and skin rash. The gastrointestinal symptoms observed might be, at least in part, related to the presence of lactose in the formulation of deferasirox. Tablets contain lactose in an approximately 1:1 ratio by weight to the active drug substance deferasirox. Mild, stable increases in serum creatinine were observed in a greater percentage of patients receiving deferasirox than those receiving deferoxamine, although the percentage of patients with serum creatinine values above the normal range was similar in both groups. A dose reduction algorithm for increased serum creatinine resulted in stabilisation or normalisation of this parameter in all patients. Reversible increases in ALT were seen in a relatively small percentage of patients, and were not associated with changes in alkaline phosphatase or bilirubin levels. Such elevations in ALT may be associated with hepatic iron toxicity itself (Jensen *et al*, 2003). However, at least one patient enroled in this trial did appear to have elevations in ALT that were caused by deferasirox administration. This conclusion was based on the isolated recurrence of elevated aminotransferase upon rechallenge with deferasirox. Drug-induced hepatitis was not confirmed because the ALT values returned to normal within a short enough timeframe, so a liver biopsy was not performed.

Although these data indicate that deferasirox was well tolerated in individuals with sickle cell disease who had normal baseline serum creatinine levels, the safety of deferasirox in sickle cell patients with abnormal serum creatinine is unknown. Further studies will be required in this regard. In addition, whereas iron chelation may be required for many years or even life-long, the duration of this study was only 1 year. Therefore, long term follow-up of patients receiving continued treatment is required, and this is currently ongoing in an extension of the present study.

Compliance with the administration of parenteral deferoxamine chelation therapy has proved challenging to all groups of patients with transfusional iron overload (Cappellini *et al*, 2006). This is particularly true for patients with sickle cell disease (Treadwell *et al*, 2005). Treatment adherence to therapy in the present study was mandated by the study protocol and was similar in both treatment groups. However, in routine clinical practice, compliance with a once-daily, oral regimen would be expected to be superior to compliance with a parenteral regimen. Such improved compliance with therapy should lead to a reduction in morbidity and mortality from iron overload.

When combined with appropriate laboratory monitoring, the availability of deferasirox as a once-daily, oral option for safe and effective chelation therapy has the potential to prevent complications of iron overload. In paediatric and adult patients with sickle cell disease this should consequently help prevent serious complications, such as stroke and organ failure, by facilitating the appropriate use of blood transfusions.

## Appendix

**Appendix I:** Participating centres and investigators.

Canada	Olivieri N, Toronto General Hospital, Toronto.
France	Bachir D, Hôpital Henri Mondor, Créteil; Bernaudin F, Centre Hospitalier Intercommunal, Créteil; de Montalembert M, Hôpital Necker, Paris.
Italy	Cappellini MD, Ospedale Maggiorre-IRCCS, Milan; Cianciulli P, Ospedale Galliera, Rome; Forni GL, Centro della Microcitemia, Ospedale Galliera, Genoa; Lombardo T, Ospedale S Bambino, Catania; Magnano C, Az. Osp. Di Rilievo Nazionale e di Alta, Cantania.
UK	Okpala I, St. Thomas Hospital, London; Porter J, University College Hospital, London.
USA	Adewoye A, Boston Medical Center, Boston; Bellevue R, New York Methodist Hospital, Brooklyn, NY; Benjamin L, Montefiore Hospital, Bronx, NY; Cataland S, Ohio State University, Columbus, OH; Clowney B, Santee Hematology/Oncology, Sumter, SC; Coates T, Children's Hospital Los Angeles, CA; Cruz J, Wake Forest University, Winston-Salem, NC; Eckman J, Emory University School of Medicine, Atlanta, GA; Frankel L, Scott & White Memorial Hospital, Temple; Freiberg A, Penn State Milton S Hershey Medical Center, Hershey, PA; Gardner R, Children's Hospital, New Orleans, LA; Giardina P, Weill Medical College of Cornell University, New York, NY; Gonzalez F, Liberty Hematology Oncology Center, Columbia, SC; Hassell K, University of Colorado Health Sciences Center, Denver, CO; Heeney M, Children's Hospital of Boston, Boston, MA; Kelly P, Children's Hospital Medical Center, Cincinnati, OH; Krishnamurthi L, Children's Hospital of Pittsburgh, Pittsburgh, PA; Kutlar A, Medical College of Georgia, Augusta, GA; Kwiatkowski J, Children's Hospital of Philadelphia, Philadelphia, PA; Labotka R, University of Illinois at Chicago, Chicago, IL; Lane P, Emory University School of Medicine, Atlanta, GA; Mathias L, Loma Linda University Medical Center, Loma Linda, CA; Mueller, BU, Texas Children's Hospital, Houston, TX; Nuss R, University of Colorado Health Science Center, Denver; Onyekwere O, Howard University, Washington, DC; Owen W, Children's Hospital of the King's Daughter, Norfolk, VA; Prasannan L, Scott and White Memorial Hospital and Clinics, Temple, TX; Rice L, The Methodist Hospital, Houston, TX; Scher C, Tulane University, New Orleans, LA; Swerdlow P, Wayne State University School of Medicine, Detroit, MI; Tebbi C, Tampa Children's Hospital at St. Joseph's, Tampa, FL; Thompson A, Children's Memorial Hospital, Chicago, IL; Vichinsky E, Children's Hospital and Research Center at Oakland, Oakland, CA; Wilson F, University of South Alabama, Mobile, AL; Wise R, Palmetto Health, Columbia, SC; Yasin Z, University of Cincinnati, Cincinnati OH.
